# Integrated proteomic and metabolomic analyses reveal testicular metabolic mechanisms underlying sperm quality in drakes

**DOI:** 10.1016/j.psj.2025.106349

**Published:** 2025-12-29

**Authors:** Chunhong Zhu, Haotian Gu, Zhicheng Wang, Weitao Song, Zhiyun Tao, Shuangjie Zhang, Huifang Li, Hongxiang Liu

**Affiliations:** Jiangsu Institute of Poultry Sciences, Yangzhou 225125, China

**Keywords:** Drake, Sperm quality, Testis, Proteomic analysis, Metabolomic analysis

## Abstract

This study aimed to analyze the testicular metabolic mechanisms associated with the sperm quality of drakes from proteomic and metabolomic perspectives. Testicular tissues were collected from 12 drakes (six with significantly high and six with significantly low sperm quality) to evaluate sperm quality parameters. Ultra-deep quantitative proteomics and ultra-performance liquid chromatography-tandem mass spectrometry were employed to analyze differences in testicular proteomics and metabolites between groups. Enrichment analysis of differentially expressed proteins (DEPs) and metabolites (DEMPs) in testicular tissues was performed to identify key pathways associated with sperm quality. In total, 153 DEPs (65 upregulated, 88 downregulated) and 98 DEMPs (15 upregulated, 83 downregulated) were identified in the testes of drakes with varying sperm quality. Functional enrichment analysis revealed that these DEPs and DEMPs were mainly involved in the key biological processes and pathways of oxidative phosphorylation, fatty acid degradation, glycerophospholipid metabolism, and purine metabolism. Notably, downregulated carnitines highlighted lipid metabolic balance and fatty acid-derived energy as critical for sperm motility, while differential expression of oxidative phosphorylation core proteins (e.g., ATP6V0C, SDHD) confirmed efficient ATP production as pivotal for sperm function. Purine metabolism and related metabolites further linked metabolic homeostasis to potential energy dysfunction or DNA damage. These findings fill gaps in avian sperm research and identify candidate targets (proteins, metabolites, and pathways) for genetic breeding to enhance avian male fertility and optimize poultry breeding programs.

## Introduction

Sperm quality is a critical determinant of the reproductive efficiency of poultry breeding that directly affects the success rate of artificial insemination and the health of offspring. Despite this importance, the molecular mechanisms underlying the variation of sperm quality remain poorly understood, especially for avian species (Asano and Tajima, 2017). Recent advancements in proteomics and metabolomics have allowed for the comprehensive identification and quantification of proteins and metabolites in reproductive tissues, revealing their roles in semen and sperm function and viability, thereby providing new insights into the molecular mechanisms underlying sperm quality (Long, 2020; Vitorino Carvalho et al., 2021).

Previous studies of mammalian species (e.g., humans, boars, and bulls) have identified key proteins (e.g., spermadhesins, antioxidant enzymes, and energy metabolism-related proteins) and metabolites (e.g., adenosine triphosphate [ATP], carnitine, and glutathione) associated with sperm motility, viability, and DNA integrity (Alipour et al., 2021; Blagojevic et al., 2024; Li et al., 2016; Pena et al., 2022; Zhang et al., 2021). In poultry, limited proteomic and metabolomic analyses have revealed seminal plasma proteins (e.g., ovotransferrin and albumin) and metabolic pathways (e.g., glycolysis and lipid metabolism) that are crucial for sperm function (Santiago-Moreno and Blesbois, 2020). A comparative proteomic study identified 55 differentially expressed proteins (DEPs) in the seminal plasma of drakes with high-quality semen versus low-quality semen. These DEPs were primarily involved in transmembrane transport, extracellular matrix structural components, and metabolic pathways, such as glycerophospholipid metabolism (Tang et al., 2022). Another study of Manila drake semen focused on the formulation of cryoprotectants and the impact on frozen semen quality, emphasizing the need for optimal conditions to maintain sperm viability (Giri et al., 2016; Telnoni et al., 2024). However, most studies have focused on chickens, leaving a significant knowledge gap regarding the sperm quality of drakes, which exhibit distinct reproductive physiology, including a higher volume but lower concentration of sperm as compared to chickens (Du et al., 2022; Ma et al., 2025).

Despite these advancements, the molecular mechanisms regulating drake semen quality, particularly at the testicular tissue level, remain unclear. Drakes exhibit unique reproductive physiology as compared to mammals, with distinct differences in semen volume and concentration (Calisto Bongalhardo, 2025). The low sperm quality of drakes has become a major constraint to the development of artificial insemination technology. Therefore, it is essential to explore the proteomic and metabolomic profiles of drake testicular tissues to identify potential biomarkers and pathways associated with sperm quality.

This study aimed to investigate the proteomic and metabolomic profiles of drake testicular tissues to elucidate the molecular mechanisms underlying semen quality. By identifying key proteins and metabolites involved in sperm function, we sought to uncover novel biomarkers and pathways associated with the sperm quality of drakes. This integrative approach will provide a comprehensive understanding of factors influencing sperm quality and fertility of drakes. The findings of this study will offer valuable insights for the development of new strategies to improve artificial insemination success rates and optimize breeding programs in the poultry industry.

## Materials and methods

### Animals and sample preparation

In total, 160 35-week-old Jinding drakes with similar body weight and genetic background were provided by Jiangsu Gaoyou Drake Development Group Co., Ltd. (Jiangsu, China). All drakes were kept in individual cages under identical temperature and lighting conditions. Throughout the experimental period, the drakes were exposed to 16-h light cycles and had *ad libitum* access to water and a standard diet. The experimental diets were formulated to meet the nutritional needs of drakes as recommended by the National Research Council of China. The sperm of all drakes was collected and four sperm viability parameters and eight sperm motility parameters were measured every other day for a total of 12 measurements. These sperm parameters were assessed using the Computer-Assisted Sperm Analysis (CASA) system (ML-500JZ; Nanning Songjing Tianlun Biotechnology Co., Ltd., Nanning, China). Testicular tissues were collected from six individuals with the highest sperm quality (G_H1-G_H6) and six individuals with the lowest sperm quality (G_L1-G_L6).

All protocols for sample collection samples were reviewed and approved by the Animal Care and Use Committee of Jiangsu Institute of Poultry Science, and conducted in accordance with established regulations and guidelines.

### Label-free-based quantitative proteomics analysis

#### Protein extraction and digestion

Proteins were isolated from drake testis samples using lysis buffer (8 M urea, 1 mM phenylmethylsulfonyl fluoride, and 2 mM ethylenediaminetetraacetic acid). The samples were lysed on ice for 5 min and then centrifuged (15,000 × *g*, 4°C, 10 min). Afterward, the supernatant was collected and the protein concentration was measured using a bicinchoninic acid assay kit (Beyotime Biotechnology, Shanghai, China).

Then, protein solution (100 μg adjusted to a volume of 200 μL with 8 M urea) was reduced with 5 mM dithiothreitol at 37°C for 45 min, followed alkylation with 11 mM iodoacetamide in the dark at room temperature for 15 min and overnight digestion at 37°C with ammonium bicarbonate solution (800 μL, 25 mM) and trypsin (3 μL; Promega Corporation, Madison, WI, USA). The pH of the digested peptide solution was adjusted to 2–3 using 20 % trifluoroacetic acid, followed by desalting with C18 resin (EMD Millipore Corporation, Billerica, MA, USA). Finally, the peptide concentration was determined using a Pierce™ Quantitative Peptide Assay Kit with standards (Thermo Fisher Scientific, Waltham, MA USA).

#### Ultra-performance liquid chromatography–tandem mass spectrometry (UPLC-MS/MS)

The digested peptides (∼200 ng) were separated within 40 min at a flow rate of 0.3 μL/min using a nanoElute UHPLC system (Bruker Daltonics GmbH & Co. KG, Bremen, Germany) equipped with a commercially available reverse-phase C18 column and an integrated CaptiveSpray Emitter (25 cm × 75 μm; inner diameter, 1.6 μm, Aurora Series with a captive spray interface adapter; IonOpticks, Fitzroy, VIC, Australia). The separation temperature was maintained by an integrated toaster column oven at 50°C. Mobile phases A and B consisted of 0.1 % formic acid in water and 0.1 % formic acid in acetonitrile, respectively. Mobile phase B was increased from 2 % to 22 % over the first 25 min, increased to 35 % over the next 5 min, further increased to 80 % over the next 5 min, and then held at 80 % for 5 min.

The LC system was coupled online to the timsTOF Pro2 open platform (Bruker Daltonics GmbH & Co. KG) via a CaptiveSpray nano-electrospray ion source. The collision energy was increased linearly as a function of mobility from 45 eV at 1/K0 = 1.3 Vs/cm^2^ to 27 eV at 1/K0 = 0.85 Vs/cm^2^. The quadrupole isolation width was set to 2 Th for m/*z* < 700 and 3 Th for m/*z* > 800.

#### Database search and quantification

The raw MS data were analyzed using the automated data-independent acquisition (DIA) proteomics data processing software suite DIA-NN (v1.8.1; https://github.com/vdemichev/DiaNN) with the library-free method. The FASTA database (16883 sequences) was used to create a spectra library with deep learning algorithms of neural networks. The MBR (match between runs) option was employed to create a spectral library from the DIA data and then reanalyzed against the library. The false discovery rate of the search results was adjusted to < 1 % at both the protein and precursor ion levels, while the remaining identifications were used for further quantification analysis. The LC-MS/MS data were searched against the *Anas platyrhynchos* (Northern mallard) proteome reference. The thresholds to identify significant DEPs were set at fold change (FC) ≥ 1.3 or ≤ 0.7692 and a probability (*p*) value < 0.05 (*t*-test). Functional enrichment analysis to identify overrepresented biological functions and pathways of the DEPs was conducted using Gene Ontology (GO) and annotated in reference to the Kyoto Encyclopedia of Genes and Genomes (KEGG).

### Metabolite analysis

#### Sample preparation and extraction

Each ground sample (20 mg) was added to a solution of methanol and water (7:3 v/v; 400 μL) containing internal standards and shaken (2500 rpm, 5 min). After cooling on ice for 15 min, the sample was centrifuged (12000 rpm, 10 min, 4°C). The supernatant (300 μL) was collected, cooled at −20°C for 30 min, centrifuged (12,000 rpm, 3 min, 4°C), and aliquoted (200 μL) for metabolite analysis by LC-MS.

#### T3 UPLC conditions

The sample extracts were analyzed using an LC-electrospray ionization (ESI)-MS/MS system (UPLC, ExionLC AD, https://sciex.com.cn/; MS, QTRAP® System, https://sciex.com/) equipped with an ACQUITY UPLC HSS T3 C18 column (1.8 µm, 2.1 mm*100 mm; Waters Corporation, Milford, MA, USA). The analytical conditions were as follows: column temperature, 40°C; flow rate, 0.4 mL/min; injection volume, 2 μL; solvent system, water (0.1 % formic acid): acetonitrile (0.1 % formic acid); solvent B gradient program, 5 % to 20 % in 2 min, increased to 60 % over the following 3 min, increased to 99 % in 1 min and held for 1.5 min, and then reduced to 5 % within 0.1 min and held for 2.4 min.

#### ESI-QTRAP-MS/MS

Triple quadrupole (QQQ) scans with linear ion trap (LIT) technology were acquired with a triple quadrupole-linear ion trap mass spectrometer (QTRAP® LC-MS/MS System; SCIEX, Framingham, MA, USA) equipped with an ESI Turbo Ion-Spray interface, operating in positive and negative ion mode and controlled by Analyst 1.6.3 software (SCIEX). The ESI source operation parameters were as follows: source temperature, 500°C; ion spray voltage, 5500 V (positive) / −4500 V (negative); ion source gas I pressure, 55 psi; gas II pressure, 60 psi; curtain gas pressure, 25.0 psi; and collision gas, high. Instrument tuning and mass calibration were performed with 10 and 100 μmol/L of polypropylene glycol solutions in QQQ and LIT modes, respectively. Multiple reaction monitoring of a specific set of transitions of the eluted metabolites was conducted.

#### Bioinformatics analysis of DEPs and differentially expressed metabolites (DEMPs)

The obtained data were annotated against the *Anas platyrhynchos* proteome reference on the UniProt website (https://www.uniprot.org/proteomes/UP000016666). The criteria for DEPs were an absolute FC≥1.3 or ≤ 0.7692 and *p*-value < 0.05 (*t*-test), while the criteria for DEMPs were a Variable Importance for Projection (VIP) score > 1 and *p*-value < 0.05 (Student's *t*-test). VIP scores were determined by orthogonal partial least squares discriminant analysis (OPLS-DA). Score plots and permutation plots were generated using the R package “MetaboAnalystR” (https://www.metaboanalyst.ca/docs/RTutorial.xhtml). The data were log transformed (log2) and mean centered before OPLS-DA. In order to avoid overfitting, permutation testing (200 permutations) was performed.

Unsupervised principal component analysis (PCA) was performed with the R function “prcomp” (https://masedki.github.io/enseignements/pca.html). The data were unit variance scaled before unsupervised PCA. The results of hierarchical cluster analysis (HCA) of the samples and metabolites are presented as heatmaps with dendrograms, while Pearson correlation coefficients (PCCs) between samples were calculated with the R function “core” (https://www.rdocumentation.org/packages/its/versions/1.1.8/topics/core) and are presented as heatmaps. Heatmaps of the HCA and PCC results were generated using the R package “ComplexHeatmap” (https://github.com/jokergoo/ComplexHeatmap). For HCA, normalized signal intensities of metabolites (unit variance scaling) are visualized as a color spectrum.

GO terms were mapped using the UniProt-GOA database (https://www.ebi.ac.uk/GOA/uniprot_release), and functional enrichment analysis of all DEPs and DEMPs was performed by the ClusterProfiler R package in reference to the KEGG. The GO terms and KEGG pathways with corrected *p*-values < 0.05 (two-tailed Fisher's exact test) were considered significant.

### Statistical analysis

Statistically significant differences between the G_H and G_L groups were determined by one-way analysis of variance. A *p*-value < 0.05 was considered statistically significant.

## Results

### Quality of drake sperm

Semen quality of 160 drakes (age, 230 days; similar body weight) was assessed using the CASA system. The results are shown in Table 1, Table 2. In total, six high-quality (G_H) samples and six low-quality (G_L) samples were selected based on significant differences in semen quality parameters, as determined by PCA of multiple parameters. There were no significant differences in body weight between the groups, however, it shown significant differences in sperm viability (motility, viability, density, and mortality rate) and motility parameters (VCL, VAP, ALH, and MAD) at *p* = 0.01 level. the results also showed the *p* value of motility parameter VSL was 0.030.

**Table 1:** Comparison of body weight and sperm viability parameters between the G_L and G_H groups.

Sample/No.	Body weight, g	Testicular volume, cm^3^	Sperm motility, %	Sperm viability, %	Sperm density, %	Sperm mortality rate, %
Total/160	1389.389 ± 126.189	165.188 ± 52.883	91.146 ± 6.791	95.908 ± 4.422	0.747 ± 0.158	4.092 ± 4.422
G_H/6	1422.167 ± 133.311	230.757 ± 47.599	97.617 ± 0.933	99.392 ± 0.513	0.938 ± 0.069	0.608 ± 0.513
G_L/6	1420.333 ± 197.988	130.529 ± 66.635	69.317 ± 8.251	81.714 ± 7.252	0.573 ± 0.218	18.286 ± 7.252
p_value	0.985	0.013	0.000	0.000	0.009	0.000

**Table 2:** Comparison of sperm motility parameters between the G_L and G_H groups.

Sample/No.	VSL, um/s	VCL, um/s	VAP, um/s	ALH, um	WOB	LIN	MAD	STR
Total/160	24.179 ± 4.229	54.050 ± 7.346	38.219 ± 5.195	15.831 ± 2.152	0.922 ± 0.041	0.448 ± 0.035	648.018 ± 190.747	0.634 ± 0.049
G_H/6	35.404 ± 5.389	71.982 ± 12.54	50.901 ± 8.866	21.083 ± 3.673	0.935 ± 0.062	0.444 ± 0.058	880.836 ± 157.019	0.627 ± 0.083
G_L/6	19.333 ± 2.112	43.423 ± 2.647	30.706 ± 1.871	12.718 ± 0.776	0.896 ± 0.032	0.451 ± 0.032	403.405 ± 127.93	0.637 ± 0.043
p_value	0.030	0.000	0.000	0.000	0.199	0.811	0.000	0.798

Note: VSL (Straight-line Velocity), um/s; VCL (Curvilinear Velocity), um/s;VAP (Average Path Velocity), um/s; ALH (Amplitude of Lateral Head Displacement), µm; WOB (Wobble); LIN (Linearity); MAD (Angular Displacement); STR (Straightness).

### Identification and comparison of DEPs and DEMPs in testicular tissues between the G_H and G_L groups

**Fig. 1:**
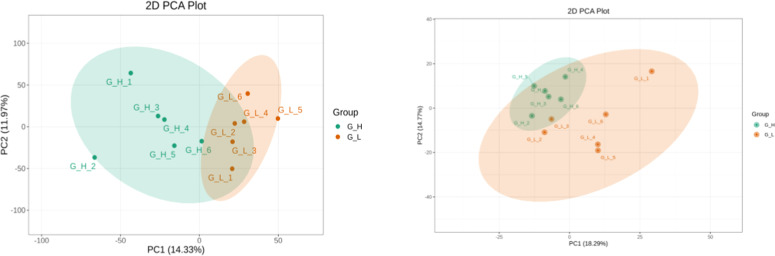
PCA of DEPs and DEMPs between the G_L and G_H groups.

Comparative proteomics and metabolomics analyses of the G_L and G_H groups were performed using the label-free proteomic approach and an LC-ESI-MS/MS system. The results revealed differences in proteins and metabolites of the testicular tissues of the G_L and G_H groups. PCA was performed to assess DEPs and DEMPs. The results are presented in Fig. 1.

**Fig. 2:**
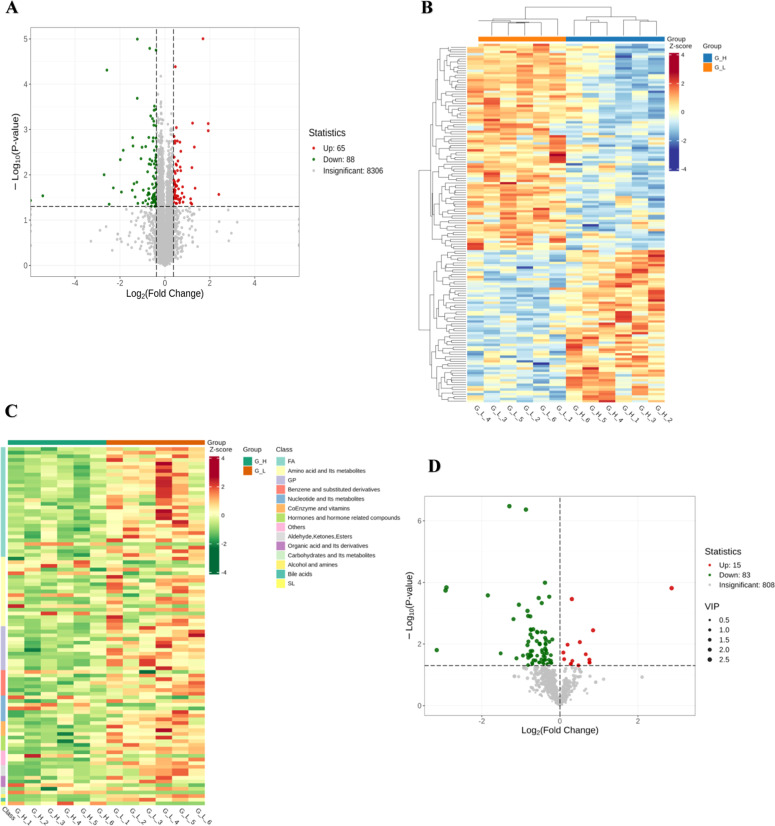
Volcano plot and clustering maps of DEPs and DEMPs between the G_L and G_H groups.

Volcano plots and clustering maps were generated of 153 DEPs in testicular tissues (65 upregulated and 88 downregulated) between the G_L and G_H groups (Fig. 2A, Table S1). VIP scores were calculated for 98 DEMPs in testicular tissues (15 upregulated and 83 downregulated) between the G_L and G_H groups (Fig. 2D, Table S2). DEMPs mainly included fatty acyls (*n* = 30), amino acids and related metabolites (*n* = 19), glycerophospholipids (*n* = 12), nucleotides and related metabolites (*n* = 7), benzene and substituted derivatives (*n* = 7), coenzymes and vitamins (*n* = 4), hormones and hormone-related compounds (*n* = 4), aldehydes, ketones, and esters (*n* = 3), organic acids and related derivatives (*n* = 3), carbohydrates and related metabolites (*n* = 2), alcohol and amines (*n* = 1), bile acids (*n* = 1), sphingolipid (*n* = 1), and others (non-classifiable) (*n* = 4). Most fatty acyls were carnitines (83.3 %, 25/30).

### Functional enrichment analysis of the characteristic DEPs and DEMPs of testes between the G_L and G_H groups

**Fig. 3:**
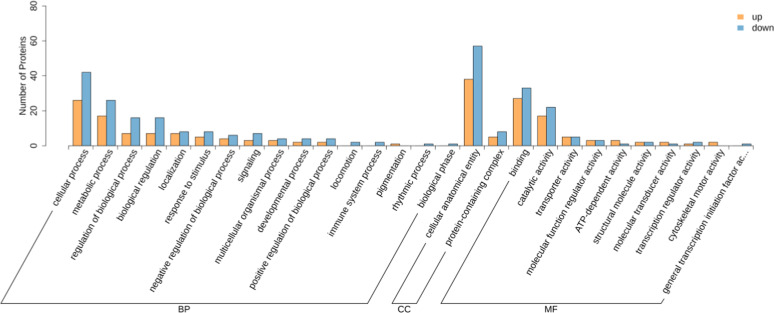
Bar chart of GO functions of DEPs in testicular tissues between the G_L and G_H groups.

GO and KEGG analyses were performed to clarify the biological functions of the DEPs. GO analysis revealed that the top enriched terms were mostly related to cellular anatomical entity, cellular process, binding, metabolic process, and catalytic activity (Fig. 3). The upregulated proteins associated with these pathways included MYO3B, MGAT5, MGST2, PIK3CB, POMK, SDHD, and ANKRD27, while the downregulated proteins included LSM1, LSM7, NUDT3, PINK1, LAMA3, and RHOG (Table S3).

**Fig. 4:**
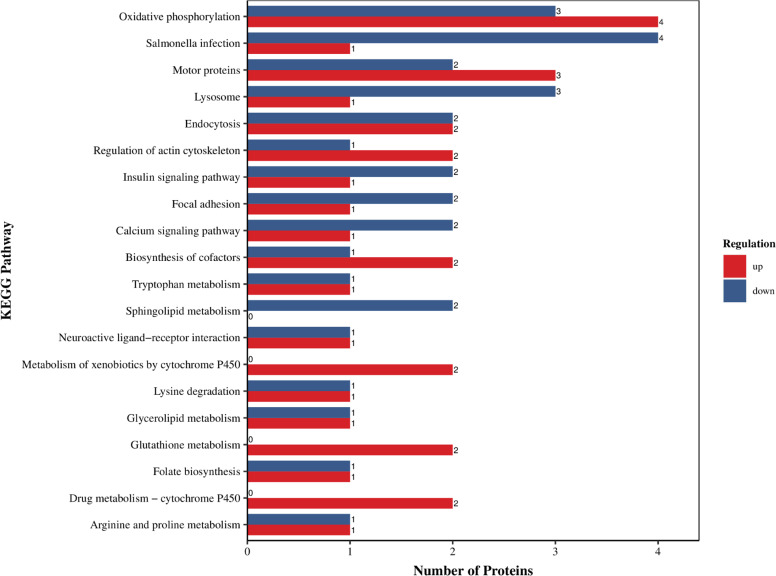
KEGG functional enrichment analysis of DEPs in testicular tissues between the G_L and G_H groups.

The results of KEGG enrichment analysis of DEPs identified a total of 79 pathways, which were primarily related to metabolism, environmental information processing, and cellular processes. The top 20 pathways are shown in Fig. 4. Among the top 20 pathways, 16 were related to metabolism of energy, amino acids, and lipids (Table S4).

**Fig. 5:**
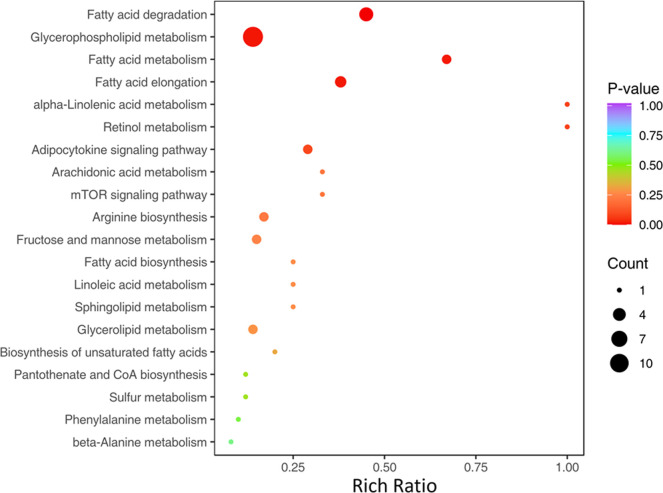
Top 20 enriched KEGG pathways of DEMPs in testicular tissues between the G_L and G_H groups.

The DEMPs were associated with 79 enriched pathways. The majority (87.88 %) of DEMPs were associated with metabolism (Fig. 5), while 12.12 % were related to environmental information processing. The top 20 enriched pathways included fatty acid degradation (*p* = 0.00059), glycerophospholipid metabolism (*p* = 0.01276), fatty acid metabolism (*p* = 0.01543), and fatty acid elongation (*p* = 0.01644) (Table S5).

### The nine-quadrant analysis of the correlation between DEPs and DEMPs

**Fig. 6:**
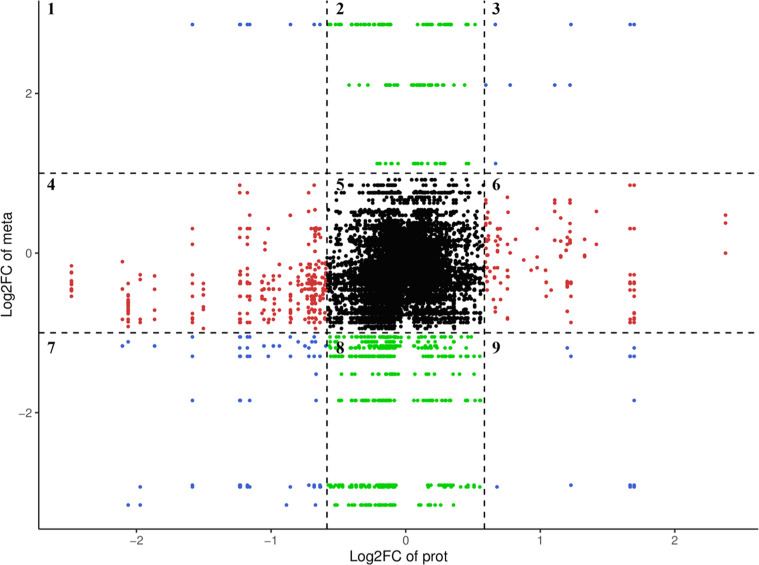
Nine-quadrant analysis of the correlation between DEPs and DEMPs in G_L and G_H groups. The x-axis represents the log₂(FC) of DEPs, while the y-axis represents the log₂(FC) of DEMPs. The chart is divided into nine quadrants by the black dashed lines, numbered 1 to 9 sequentially from left to right and top to bottom. Quadrants 1 & 9: The protein and metabolite show discordant differential expression (one up- and the other down-regulated), indicating an inverse regulatory trend; Quadrants 2, 4, 6, 8: Only one component (either the protein or the metabolite) is differentially expressed while the other remains unchanged; Quadrant 5: Neither the protein nor the metabolite is differentially expressed in this comparison; Quadrants 3 & 7: The protein and metabolite show concordant differential expression (both up- or both down-regulated), indicating a positive correlation.

The metabolite maltitol and the proteins RAB11FIP1, SSR3, YIPF6, and LOC113845425 were simultaneously upregulated, grouping in phase 3 (Fig. 6). Meanwhile, 7-ketocholesterol, l-lysine-l-threonine, and other downregulated proteins (Table S6) were grouped in phase 7. The nine-quadrant diagram revealed that the metabolite maltitol was upregulated, while the proteins BET1L, CERS5, LOC101790581, LSM7, RBM8A, and SYNDIG1 were downregulated in phase 1. In the nine-quadrant diagram, the DEMPs that showed similar or opposite trends to the proteins mainly included maltitol, 2,6-di‑tert‑butyl‑1,4-benzoquinone, carnitine C20:1-OH, 7-ketocholesterol, all-trans-13,14-dihydroretinol, hyodeoxycholic acid, deoxyguanosine, l-lysine-l-threonine, lipopolysaccharide (20:1), and 3-deoxyguanosine, which were distributed in phases 1 and 9 (Table S6).

**Fig. 7:**
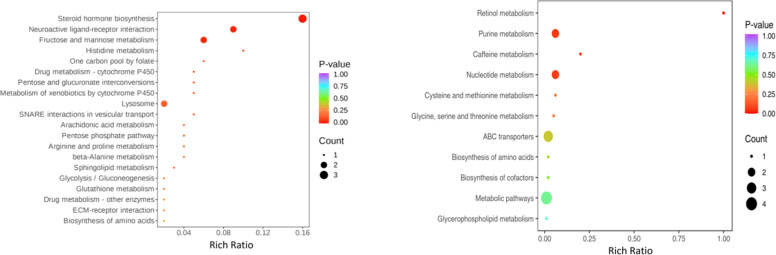
KEGG pathways of proteins (left) and metabolites (right) in quadrants 1, 3, 7, and 9 in the nine-quadrant diagram of testicular tissues between the G_L and G_H groups.

The proteins and metabolites in phases 1, 3, 7, and 9 of the nine-quadrant diagram were subjected to KEGG enrichment analysis (Fig. 7). The results (Fig. 6) revealed that the proteins ALDOB and LOC101790581, and the metabolites deoxyguanosine, 3-deoxyguanosine, and l-cystathionine were co-enriched in purine metabolism and biosynthesis of amino acids. However, these proteins and metabolites were not differentially expressed in testicular tissues between the G_L and G_H groups.

### Commonly enriched KEGG pathways as determined by proteomics and metabolomics

**Fig. 8:**
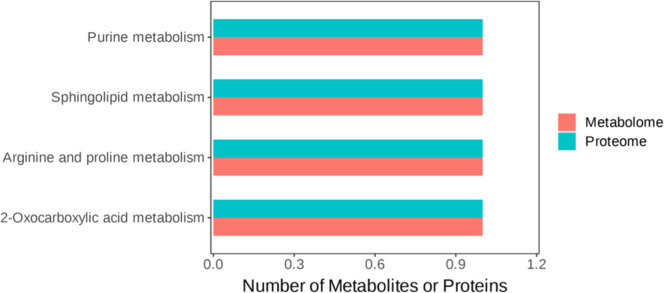
KEGG pathway co-enrichment analysis based on proteomic and metabolomic data of testicular tissues from the G_L versus G_H comparison. The horizontal axis lists the commonly enriched KEGG pathways, and the vertical axis shows the corresponding differentially expressed proteins (DEPs, blue) and metabolites (DEMPs, red) associated with each pathway.

A bar chart of the commonly enriched KEGG pathways as determined by proteomics and metabolomics is presented in Fig. 8. Four pathways were enriched (purine metabolism, sphingolipid metabolism, arginine and proline metabolism, and 2-oxocarboxylic acid metabolism). The enriched DEPs (LOC101790581, CERS5, AGMAT, and AADA) and DEMPs (T5′-Adenylyl sulfate, sphinganine (d18:0), l-arginine, and N-acetylornithine) are shown in Table 3 and Table S7.

**Table 3:** DEPs and DEMPs of drake testicular tissues involved in pathways associated with sperm quality as determined by metabolomics and proteomics.

Pathways	Metabolites	Fold change (Log2FC)	Protein	Fold change
2-Oxocarboxylic acid metabolism	N-Acetylornithine	−0.521	p.AADAT	0.477
Arginine and proline metabolism	L-Arginine	−0.521	p.AGMAT	0.179
Sphingolipid metabolism	SPH (d18:0)	0.511	p.CERS5	0.624
Purine metabolism	5′-Adenylyl sulfate (APS)	−0.466	p.LOC101790581	0.373

## Discussion

Semen quality of drakes directly affects the fertilization rate, hatching rate, and egg production efficiency. High-quality semen can reduce embryo mortality and improve chick health (Me et al., 2019). There were significant differences in semen quality among the different breeds of male drakes in this study. Therefore, from a genetic perspective, an elucidation of the genetic mechanisms could improve the semen quality and breeding performance of drakes.

In this study, the CASA system was employed to assess the semen quality of Jinding drakes. Notably, most studies of semen quality are performed for artificial insemination of humans and livestock, while investigations of birds have been relatively limited. The semen quality of Jinding drakes was determined by analyzing parameters associated with viability (motility, viability, density, and mortality rate) and motility (VSL, VCL, VAP, ALH, WOB, LIN, MAD, and STR). For analysis, two groups (G_L and G_H) were established of individuals with significant differences in semen quality, other than the parameters STR, LIN, and WOB. The testicular tissues of the selected individuals were subjected to proteomic and metabolomic sequencing to reveal DEPs and DEMPs that regulate semen quality.

In total, 153 DEPs and 98 DEMPs were detected in testicular tissues between the G_H and G_L groups. Notably, among the 98 DEMPs, 25 (25.51 %) were carnitines, and all were downregulated. Carnitine and acetyl carnitine are core participants in lipid metabolism, functioning to transport long-chain fatty acids into the mitochondria for *β*-oxidation to generate ATP, which supports sperm motility (Amaral et al., 2014; Jeulin and Lewin, 1996; Terner, 1960). By promoting fatty acid oxidation for energy production and reducing peroxidation of polyunsaturated fatty acid, carnitine plays a key role in regulating lipid metabolism, maintaining metabolic balance, and minimizing cellular damage from lipid peroxidation. High concentrations of carnitine are essential for sperm maturation and motility. Hence, carnitine supplementation can significantly improve the semen quality of poultry (Rizk et al., 2019). For instance, dietary supplementation of carnitine at 500 mg/kg was reported to increase the concentration and reduce lipid peroxidation of sperm of the White Rock rooster (Neuman et al., 2002). Acetylcarnitine, the active form of carnitine, primarily promotes fatty acid transport and oxidation to provide cellular energy. In testicular tissues, acetylcarnitine is involved in regulating energy metabolism and antioxidant defense mechanisms during spermatogenesis (Mateus et al., 2023). Sperm and seminal plasma metabolites have been identified as potential indicators of semen quality, and l-acetylcarnitine is a biomarker of male fertility (Lombo et al., 2021). However, acetylcarnitine was not differentially expressed in the present study. Nonetheless, carnitines were negatively correlated with most semen parameters, except for LIN and STR, and the correlation coefficients of most parameters were less than −0.5 (Table S8). The impact of downregulated carnitine expression in the G_L group was mainly reflected as decreased motility.

In the present study, functional analysis was conducted to reveal the biological implications of DEPs and DEMPs between the G_L and G_H groups. The results showed that most of the DEPs in testicular tissues were mainly enriched in the GO terms cellular anatomical entity, cellular process, binding, metabolic process, and catalytic activity. KEGG enrichment of the DEPs revealed that the vast majority of pathways were associated with metabolism, and many were involved in the oxidative phosphorylation pathway. Sperm motility relies on ATP hydrolysis, and both glucose and pyruvate can support ATP synthesis in sperm. However, the ATP concentration generated by oxidative phosphorylation is significantly higher than the amount produced by the glycolysis pathway (Mookerjee et al., 2017). After treatment with the metabolic inhibitors 3-monochloropropane-1,2-diol, which inhibits glycolysis, and carbonyl cyanide 3-chlorophenylhydrazone, which inhibits oxidative phosphorylation, sperm motility and ATP levels decreased synchronously, confirming the direct association between energy supply and motility (Setiawan et al., 2024). In this study, the DEPs ATP6V0C, ATP6V1C2, LOC101793848, LOC101800937, NDUFA4L2, NDUFB1, and SDHD were enriched in the oxidative phosphorylation pathway. ATP6V0C is an important subunit of vacuolar-type ATPase, which is involved in regulating pH across membranes, endocytosis, substance transport, and neurotransmitter release (Tian et al., 2022). NDUFA4L2 (NADH dehydrogenase α subcomplex 4L2) is a regulatory subunit of mitochondrial respiratory chain complex I, which stabilizes production of reactive oxygen species (ROS) under hypoxic conditions and is highly expressed in pericytes (Mesa-Ciller et al., 2023; Xu et al., 2020). NDUFB1 (a subunit of complex I) and SDHD (subunit of succinate dehydrogenase) are core proteins of the mitochondrial electron transport chain, participating in ATP generation and ROS metabolism (Hou et al., 2019; Li et al., 2020). The DMEPs were associated with fatty acid degradation, glycerophospholipid metabolism, fatty acid metabolism, and fatty acid elongation, which were all significantly enriched. These results are highly similar to those of DMPs. Fatty acid metabolism plays crucial roles in energy production, serving as a major fuel source for cells through *β*-oxidation to generate acetyl-CoA, which enters the tricarboxylic acid cycle to produce ATP (Ehara et al., 2015).

KEGG analysis found that the purine metabolism pathway was enriched. Sperm motility relies on ATP, which is largely produced by purine metabolism, and 5′-adenylyl sulfate (APS), an important intermediate in purine metabolism, is catalyzed by ATP sulfurylase (EC 2.7.7.4), which converts ATP and inorganic sulfate into APS and pyrophosphate (Venkatachalam et al., 2025). Abnormal purine metabolism can increase production of ROS, thereby triggering oxidative stress that damages sperm membranes and DNA (Soflaee et al., 2022). Moreover, accumulation of deoxyguanosine/3-deoxyguanosine, which are by-products of purine metabolism, may reflect sperm DNA damage or abnormal energy metabolism (He et al., 2024). ALDOB (aldolase B) is a key enzyme in glycolysis that indirectly regulates synthesis of purine nucleotides, thereby affecting the energy supply for sperm motility (Violante et al., 2025), although there was no difference in ALDOB content between the two groups

In summary, this study explored the molecular mechanisms underlying the sperm quality of Jinding drakes to improve avian reproduction via genetic selection. The downregulation of carnitines and differential expression of oxidative phosphorylation proteins (e.g., ATP6V0C, NDUFA4L2, and SDHD) highlight the critical roles of lipid metabolic balance and efficient ATP production. Therefore, supporting mitochondrial energy metabolism through nutritional or management strategies, such as dietary supplementation with l-carnitine or optimization of fatty acid composition, could be beneficial for improving sperm motility. Additionally, the identified DEPs and DEMPs, including specific carnitines and key metabolic proteins, serve as candidate biomarkers for genetic selection, enabling the breeding of drakes with inherently superior sperm metabolic capacity. Future research could focus on validating these biomarkers and optimizing dietary formulations that target the fatty acid degradation, glycerophospholipid metabolism, and purine metabolism pathways highlighted in this study.

## CRediT authorship contribution statement

**Chunhong Zhu:** Writing – review & editing, Writing – original draft, Investigation, Funding acquisition, Conceptualization. **Haotian Gu:** Methodology, Formal analysis, Data curation. **Zhicheng Wang:** Writing – original draft, Validation, Data curation. **Weitao Song:** Writing – review & editing, Writing – original draft, Formal analysis, Data curation, Conceptualization. **Zhiyun Tao:** Writing – original draft, Validation, Formal analysis. **Shuangjie Zhang:** Writing – original draft, Data curation. **Huifang Li:** Writing – review & editing, Funding acquisition. **Hongxiang Liu:** Writing – review & editing, Writing – original draft, Supervision, Methodology, Data curation.

## Disclosures

The authors have no conflict of interest to declare.

## Appendix Supplementary materials

Table S1. List of DEMPs. Table S2. List of DEPs. Table S3. GO terms of DEPs. Table S4. KEGG enrichment analysis of DEPs. Table S5. KEGG enrichment analysis of DEMPs. Table S6. List of DEPs and DEMPs in the nine-quadrant diagram. Table S7. KEGG enrichment analysis of DEPs and DEMPs. Table S8. Correlation analysis of carnitines and semen parameters. Image, application 1

## Footnotes

Supplementary material associated with this article can be found, in the online version, at doi:10.1016/j.psj.2025.106349.
